# Detraining Slows and Maintenance Training Over 6 Years Halts Parkinsonian Symptoms-Progression

**DOI:** 10.3389/fneur.2021.737726

**Published:** 2021-11-19

**Authors:** Tibor Hortobágyi, Dávid Sipos, Gábor Borbély, György Áfra, Emese Reichardt-Varga, Gergely Sántha, Ward Nieboer, Katalin Tamási, József Tollár

**Affiliations:** ^1^Center for Human Movement Sciences, University Medical Center Groningen, University of Groningen, Groningen, Netherlands; ^2^Somogy County Kaposi Mór Teaching Hospital, Kaposvár, Hungary; ^3^Department of Sport Biology, Institute of Sport Sciences and Physical Education, University of Pécs, Pécs, Hungary; ^4^Division of Training and Movement Sciences, University of Potsdam, Potsdam, Germany; ^5^Faculty of Health Sciences, Doctoral School of Health Sciences, University of Pécs, Pécs, Hungary; ^6^Faculty of Health Sciences, Department of Medical Imaging, University of Pécs, Pécs, Hungary; ^7^Departments of Epidemiology and Neurosurgery, University Medical Center Groningen, University of Groningen, Groningen, Netherlands; ^8^Digital Development Center, Széchényi István University, Györ, Hungary

**Keywords:** follow-up, sensorimotor training, balance training, posture, quality of life

## Abstract

**Introduction:** There are scant data to demonstrate that the long-term non-pharmaceutical interventions can slow the progression of motor and non-motor symptoms and lower drug dose in Parkinson's disease (PD).

**Methods:** After randomization, the Exercise-only (E, *n* = 19) group completed an initial 3-week-long, 15-session supervised, high-intensity sensorimotor agility exercise program designed to improve the postural stability. The Exercise + Maintenance (E + M, *n* = 22) group completed the 3-week program and continued the same program three times per week for 6 years. The no exercise and no maintenance control (C, *n* = 26) group continued habitual living. In each patient, 11 outcomes were measured before and after the 3-week initial exercise program and then, at 3, 6, 12, 18, 24, 36, 48, 60, and 72 months.

**Results:** The longitudinal linear mixed effects modeling of each variable was fitted with maximum likelihood estimation and adjusted for baseline and covariates. The exercise program strongly improved the primary outcome, Motor Experiences of Daily Living, by ~7 points and all secondary outcomes [body mass index (BMI), disease and no disease-specific quality of life, depression, mobility, and standing balance]. In E group, the detraining effects lasted up to 12 months. E+M group further improved the initial exercise-induced gains up to 3 months and the gains were sustained until year 6. In C group, the symptoms worsened steadily. By year 6, levodopa (L-dopa) equivalents increased in all the groups but least in E + M group.

**Conclusion:** A short-term, high-intensity sensorimotor agility exercise program improved the PD symptoms up to a year during detraining but the subsequent 6-year maintenance program was needed to further increase or sustain the initial improvements in the symptoms, quality of life, and drug dose.

## Introduction

Parkinson's disease (PD) impairs mobility, cognition, and quality of life (1–3). The drugs are still the primary symptom-moderators in people with PD (PwPD), as the lesion surgeries and deep brain stimulation cannot halt the progression of the underlying neurodegenerative processes (4). Physical exercise has been also used as an adjuvant to the drugs to reduce the motor and non-motor PD-symptoms and improve quality of life (2, 3, 5–19). The potency of exercise is revealed by the 21 year delay in the clinical manifestation of PD in the physically active individuals (20). In the animal models of PD, the protective effects by exercise might be related to neuroplasticity, neuroprotection, and neuro-regeneration especially at high intensities (14). PD impairs balance and postural control. When the healthy humans perform the balance exercises, the brainstem, cerebellum, basal ganglia, thalamus, and selected cortical regions become strongly and preferentially activated (21). These activation patterns provide a mechanistic basis for the symptom-reducing effects of exercise. When exercise is enriched with multi-sensory stimuli, the improvements in the motor and non-motor symptoms can be long-lasting (6, 22, 23). Indeed, high-intensity exercise can enhance the processes beyond those impacted by levodopa (L-dopa) medications (7, 9, 16, 17, 24–29). Because improvements in the symptoms outlast the exercise period during detraining, exercise might have the potential to slow not only symptom- but disease-progression (7). Accordingly, therapy-intensity predicted the length of hospital stay, hospital readmission, and functional improvements (9). However, the results are inconsistent, as improvements in the symptoms can still be independent of exercise duration, frequency, and intensity even if PwPD are of the same age, gender, and disease stage. To illustrate, the high frequency exercise can unfavorably affect the functional outcomes (30).

Thus, there is a need to demonstrate that high-intensity is in fact not harmful in moderating the disease-symptoms in PwPD and to document how long the beneficial effects of exercise last when it is stopped. Further, it is unclear if exercise is continued as a maintenance program, it would in fact prolong symptom-moderation at a functionally meaningful level (2, 18, 31, 32). While PwPD were followed without an intervention for as long as 41 years (33), the symptom and drug-dose modifying effects of long-lasting exercise maintenance are unknown. In 44 cohorts of ~15,000 PwPD, exercise was examined as a disease modifier intervention only in 11% of PwPD with median of 5 years of follow-up but without a maintenance program (34). Even if an exercise maintenance program ensued, it included only motor but not the clinical outcomes (13, 35) and was conducted at a low intensity even though the evidence suggests that exercise intensity can preserve the training effects during detraining and maintenance can heighten such effects (36).

This study aimed to determine the immediate and lasting effects of a 3-week-long, high-intensity, and high-frequency Exergaming agility program with and without a 6-year-long high-intensity Exergaming agility maintenance program, on the motor and clinical symptoms in PwPD. Based on our previous experience and the extant data, we expected that PwPD would tolerate the short-term 3-week initial high-intensity Exergaming agility program which would produce the favorable and lasting effects during detraining. In addition, we expected that the maintenance program would further slow the symptoms-progression and reduce the increase in L-dopa equivalent levels (31, 32).

## Methods

### Design and Patients

**Figure 1:**
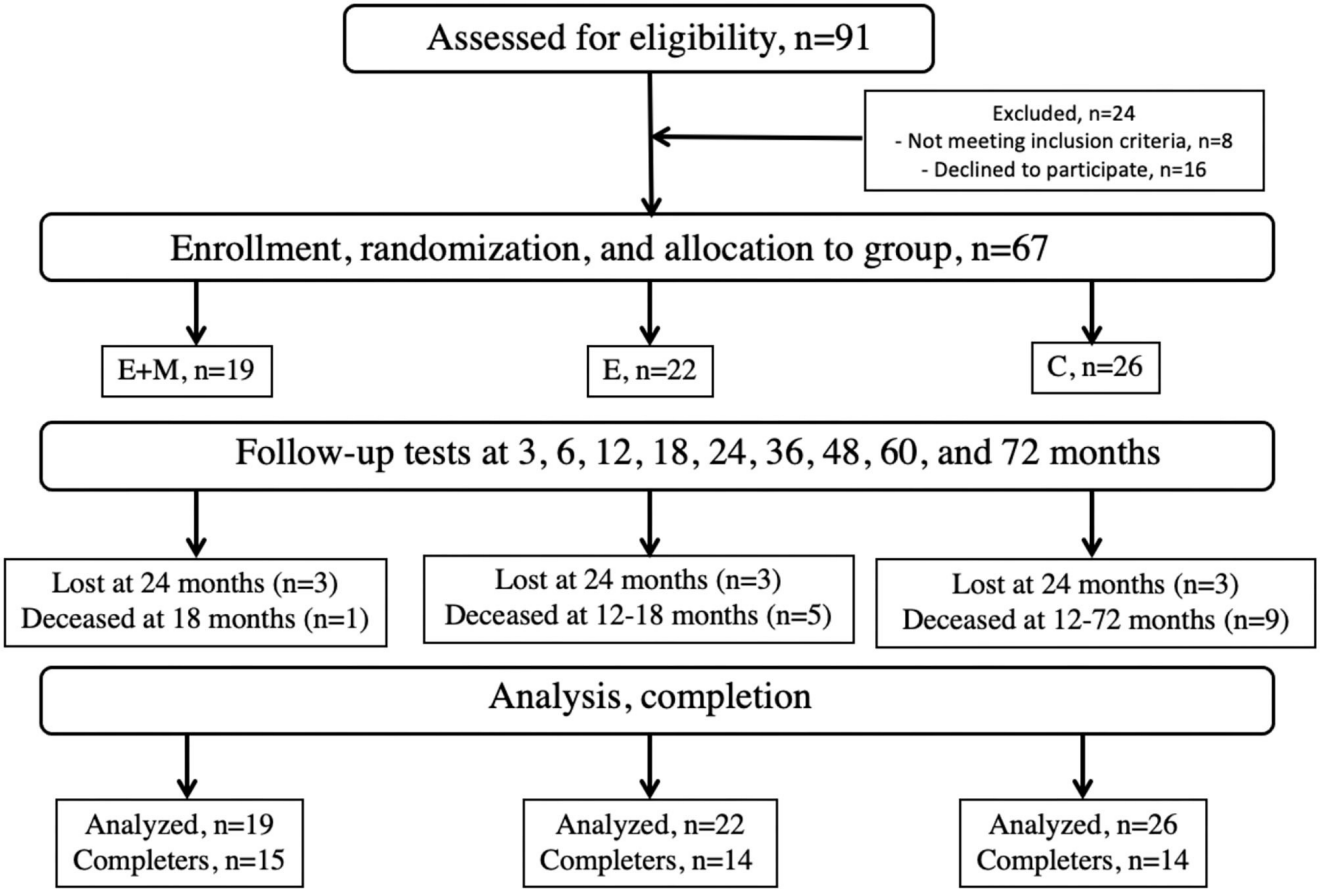
Consort diagram. E, Exergaming agility exercise program for 3 weeks, E+M, Exergaming agility exercise program for 3 weeks followed by a three times weekly Exergaming exercise Maintenance program for 6 years, and C, Control, no exercise and no maintenance.

The patients with PD in this three-group randomized clinical trial, met the UK Brain Bank criteria and had Hoehn-Yahr score of 2–3. A preliminary screening identified 91 patients from the hospital medical records. Some patients did not meet the inclusion criteria (*n* = 8) and others declined to participate (*n* = 16). The remaining 67 patients were randomized to: Exercise + Maintenance group (E + M, *n* = 19, 11M); Exercise only no maintenance group (E, f*n* = 22, 9M), and to a no exercise and no maintenance control group (C, *n* = 26, 15M; Figure 1; Table 1). The principal investigator drew a colored ribbon from a covered box and attached a colored ribbon to the folder of each patient to randomize. For a 2-year period preceding the start of the study, none of the patients were enrolled in rehabilitation. After the baseline assessment, E+M and E completed a 3-week-long agility Exergaming exercise program, which was followed by 11 assessments of all the patients at 3, 6, 12, 18, 24, 36, 48, 60, and 72 months. The M-program lasted 72 months. The wait-listed patients in C had the opportunity to enroll in the exercise program after the trial. After the 3-week-long initial exercise intervention, the patients in E and C were not enrolled in an exercise or maintenance program for the 72-month-long follow-up period. All the patients followed the neurologist-prescribed medication schedule and the M-program was continued even if changes had occurred in this schedule.

**Table 1:** The characteristics of the patients at baseline.

	**E** **+** **M**, ***n*** **=** **19 (11 M)**		**E**, ***n*** **=** **22 (9 M)**		**C**, ***n*** **=** **26 (15 M)**		**All**, ***n*** **=** **67 (35 M)**	
**Variables**	**Mean**	**±SD**	**Mean**	**±SD**	**Mean**	**±SD**	**Mean**	**±SD**
Age, y	67.5	3.91	68.1	3.53	67.8	3.84	67.8	3.71
Height, cm	173.8	6.56	172.6	4.43	174.3	5.84	173.6	5.61
Mass, kg	75.4	11.32	75.6	8.23	78.6	10.30	76.7	9.95
BMI kg·m^−2^	24.9	2.65	25.4	2.13	25.8	3.10	25.4	2.68
MMSE	27.1	1.22	27.1	1.02	27.0	1.00	27.1	1.06
PD years	6.5	2.7	6.8	1.59	7.0	2.46	6.8	2.25
Hoehn–Yahr stage	2.5	2.00	2.4	2.00	2.4	2.00	2.4	2.00
L-dopa eq., mg·d^−1^	762.5	349.36	857.5	302.84	821.2	289.75	816.5	309.32
MDS-UPDRS M-EDL	20.0	5.25	19.3	4.65	19.2	5.77	19.4	5.21
**PDQ-39**								
Mobility	17.9	6.45	16.6	4.23	6.2	8.04	16.8	6.48
Activities of daily living	9.2	4.25	9.2	2.35	8.3	4.44	8.9	3.79
Emotions	6.3	3.11	6.7	2.59	7.8	4.06	7.0	3.38
Stigma	5.1	1.90	5.6	1.62	5.5	2.98	5.4	2.29
Social	1.5	1.61	2.0	1.59	1.6	1.77	1.7	1.66
Cognition	4.6	2.36	5.1	2.10	4.5	2.87	4.7	2.48
Communication	2.6	1.92	2.5	2.11	2.3	1.64	2.4	1.86
Bodily discomfort	4.1	1.87	4.0	1.50	4.6	2.06	4.2	1.83
Sum of subitems	51.1	16.99	51.8	8.96	50.9	21.76	51.3	16.84
BDI	19.3	5.60	13.6	3.59	16.8	9.53	16.4	7.21
SE ADL, %	69.5	17.79	65.5	8.00	67.3	15.89	67.3	14.31
EQ-5D VAS, mm	64.5	13.73	69.1	8.11	63.9	11.91	65.8	11.47
**EQ-5D**								
Mobility	3.2	0.79	3.0	0.65	3.5	0.58	3.2	0.70
Self-care	2.8	0.71	2.8	0.61	3.1	0.63	2.9	0.65
Usual activities	2.4	0.76	2.5	0.51	2.5	0.81	2.4	0.70
Pain	2.5	0.77	2.5	0.67	2.6	0.75	2.5	0.72
Anxiety	3.3	0.58	2.9	0.71	3.2	0.63	3.1	0.66
Sum of subitems	14.2	2.43	13.5	1.71	14.9	2.20	14.2	2.17
TUG, s	17.0	3.81	16.1	3.54	18.3	3.81	17.2	3.78
**COP path, mm**								
Wide stance, EO	18.9	12.52	14.8	6.93	17.5	10.81	17.0	10.25
Wide stance, EC	27.1	16.26	20.5	8.89	17.6	7.79	21.2	11.63
Narrow stance, EO	25.9	14.23	23.5	9.01	23.2	8.16	24.1	10.37
Narrow stance, EC	26.7	12.77	25.6	7.32	25.7	8.46	25.9	9.43

*E + M, 3 weeks of intense agility Exercise program followed by an exercise Maintenance program, and by periodic assessments for 72 months.*

*E, 3 weeks of intense agility Exercise program, no Maintenance program, periodic assessments for 6 years.*

*C, no Exercise, no Maintenance program, periodic assessments for 72 months.*

*BMI, body mass index; MMSE, mini mental state examination; PD years, number of years since Parkinson's disease diagnosis; MDS-UPDRS M-EDL, Movement Disorder Society Unified Parkinson's Disease Rating Scale, Motor Experiences of Daily Living; L-dopa eq., L-dopa equivalent; BDI, Beck Depression Inventory (0–20, lower value less depression); SE ADL, Schwab & England Activities of Daily Living Scale (PD) (0–100, 100 denoting no mobility disability); EQ-5D, EuroQol five-dimension health-related quality of life questionnaire; VAS, visual analog scale; TUG, timed up and go test; COP, center of pressure; EO, eyes open; EC, eyes closed.*

*Median*.

In an initial screening, the patients completed: a language-validated version of Movement Disorder Society Unified Parkinson's Disease Rating Scale, Motor Experiences of Daily Living (MDS-UPDRS-M-EDL, i.e., M-EDL), a full neurological exam, and a mobility evaluation. In a separate visit, a neuropsychologist evaluated cognitive function of the patients. The neurologist and neuropsychologist were blind to the group assignments. The exclusion criteria were: MRI-based brain abnormalities; Mini Mental State Examination score <24; a Beck Depression Inventory (BDI) score >40; severe cardiac disease; uncontrolled diabetes; a history of stroke; traumatic brain injury; seizure disorder; and past or current deep brain stimulation, vestibular/visual dysfunction limiting locomotion or balance, or current participation in a self-directed or formal group exercise program. All the patients remained “on” medication that the patients took before exercise or assessment.

The assessors included two experienced physical therapists and a physical therapy assistant who were blind to the group assignments of the patients. In the familiarization session, the patients practiced each physical test and watched the Xbox Kinect Exergaming programs. The patients gave written informed consent to participate in the study. The Ethics Committee of University Hospital approved the study protocol (IKEB2020/05) and the trial was registered (NCT04559997).

### Outcome Measures

The primary outcome was M-EDL, which is sensitive to the changes in a wide array of the PD symptoms (37). The changes in M-EDL over 3.1 points are clinically meaningful (38). One of the two physical therapists, blinded to the group assignment of the patients, administered this test every time to every patient in person, to assess the motor signs of PD.

The secondary outcomes were: L-Dopa equivalents (mg·d^−1^) were computed to determine if interventions reduced the required dose; Schwab and England Activities of Daily Living Scale (SE ADL); EuroQol five-dimension health-related quality of life questionnaire (EQ-5D); the Parkinson's Disease Questionnaire (PDQ-39, clinically meaningful minimal change: 4.7 points) (39); the BDI measured depression, and the timed up and go test (TUG) quantified mobility. Postural stability was quantified by sway magnitude, measured on a force platform while standing with the eyes open in a wide and narrow stance and the eyes closed in a wide and narrow stance, in this order, for 20 s each. The outcome was the 3D path of the center of pressure in centimeters. The patients were asked to keep an exercise and weekly symptom log but we did not systematically assess the adverse events.

### Intervention

The Exergaming program comprised a high-intensity and high-frequency (5 sessions/week) agility intervention, detailed and illustrated by using the video clips (22). Briefly, E+M and E completed 15, 1-h-long, sessions over 3 weeks. Three therapists delivered the program by having the patients exercise in small groups at individual times in the physical therapy gym of the hospital. The therapists demonstrated the exercises, mingled among the patients on the exercise floor to closely supervise and spot for safety. The patients exercised without shoes on a 26-mm thick Theraband-carpeted floor. A warm-up of 10 min was followed by: (1) a 20-min block of sensorimotor and visuomotor agility training; (2) a 20-min block of sensorimotor agility training using the X-box virtual reality exergame (Microsoft Xbox 360 core system with Kinect, Microsoft Corporation, WA, USA) (40), and (3) a 10-min-long cool down. The sensorimotor and visuomotor agility training included: (1) gait training, (2) coordination training, (3) posture training with and without the augmented sensory inputs, (4) balance exercises with and without a peer, assistive devices, height stimuli, surface modifications, postural changes, shifts between tasks, and directional changes, (5) body scheme exercises, and (6) posture-corrective exercises. The exercise dosing, surface manipulations, task numbers, task types, feedback, and other methods to increase and manipulate the motor and sensory stimuli, such as the sophisticated use of the X-box virtual reality exergaming. The patients kept an exercise log to record the symptoms and fatigue. The attendance was recorded (41). Time devoted to the Exergaming and non-Exergaming routines was equal. While not measured in the present study, during such an exercise program, the average heart rate was ~121 b·min^−1^ or about 80% of the age-predicted maximum heart rate and the rate of perceived exertion was “somewhat hard/hard” or ~14 on the 20-point Borg scale (42).

### Maintenance Program

After the 3-week-long, high-intensity E, E+M continued the M-program 3 × /week for 72 months in the physical therapy gym of the hospital using the same exercises used in the 3-week exercise program. The three therapists supervised each small-group session at about the same time of the day for 1 h. The aim of the maintenance program was to determine if the patients could endure a high-intensity rehabilitation program for an extended time-period and if such a program could slow the symptom-progression.

### Control Group

This group did not participate in the E nor in the M program. We instructed these patients to continue their habitual activities. Thus, they participated in their normal social activities outside the study. They participated in the periodic testing sessions (Figure 1) and received experimental (social) attention at those times but did not receive extra social attention for what E and E + M received during the E and E + M programs.

### Statistical Analyses

The data are expressed as mean ± SD and the *CI*s. All the values were recorded for each patient at each time point. Because the PD drugs are not adjusted over a short time-period, the L-dopa values at 3-week and 3-month follow-up were imputed with the baseline value. The analyses were performed using the built-in XT (43) and the user-defined jmxtstcox package in Stata 16 (StataCorp LLC, TX, USA) (44). For each outcome, the longitudinal linear mixed effects modeling of a variable was fitted with maximum likelihood estimation using the patients as random-intercepts and each combination of treatments and time, the baseline values and additional covariates of age, sex, PD years, body mass index (BMI), L-dopa equivalent, PDQ, BDI, SE ADL, EQ-5D, EQ-5D visual analog scale (VAS), TUG, and four measures of sway as the fixed effects (45). The critical element of this modeling was the Group (E+M, E, and C) by Time (10 time points) interaction, tested by the likelihood ratio and by the coefficients, *z*, and *p*-values (95% *CI*s) for specific comparisons among the groups at the given follow-up moments. As a sensitivity analysis, we also combined the longitudinal linear mixed effects modeling with a semiparametric Cox regression to model survival by assuming the shared random intercepts (46). These analyses suggested that the time course trajectories based on the observed data and the longitudinal linear mixed effects modeling were robust so that adjusting the observed data for the losses to follow-up missing data and deaths, in addition to the respective baseline measure and covariates as done by the longitudinal linear mixed effect model, did not affect the treatment effects.

In a separate analysis, we compared those patients at the baseline who deceased over the 72 months with those who completed the trial. For this analysis, the variables not normally distributed were transformed. The comparisons were done with an unpaired *t*-test or Mann–Whitney *U*-test in the SPSS v25 (IBM, NY, USA).

The changes over time and the differences between the groups were further characterized by Cohen's effect sizes. We further quantified the within-group changes over time and between-group differences by Cohen's effect sizes (small: 0.20; moderate: 0.50; and large: 0.80). We computed Pearson's correlations between the changes in the primary and secondary outcomes to explore the potential mechanistic links underlying the improvements in the mobility and clinical symptoms of the patients. The level of significance was set at *p* < 0.05 and adjusted by Bonferroni correction for multiple comparisons in Table 2.

**Table 2:** The comparisons of the patients who completed and those who deceased during the 6-year-long study.

	**Completers, n=52**		**Deceased, n=15**			
**Variables**	**Mean**	**±SD**	**Mean**	**±SD**	***p***	***d***
Age, y	67.5	3.68	68.9	3.73	0.218	0.05
Mass, kg	76.3	9.34	77.9	12.1	0.648	0.13
BMI kg·m^−2^	25.4	2.57	25.6	3.08	0.824	0.07
**PD years**	**6.4**	**2.05**	**8.3**	**2.34**	0.005*	**0.72**
L-dopa e., mg·d^−1^	822.9	304.92	794.3	334.18	0.672	0.13
**MDS-UPDRS M-EDL**	**18.7**	**4.93**	**21.9**	**5.56**	**0.035**	**0.63**
PDQ-39 index score	48.5	14.48	60.9	21.08	0.648	0.13
BDI*	15.6	6.65	19.5	8.43	0.078	0.52
SE ADL, %	68.5	14.6	63.3	12.91	0.174*	0.32
EQ-5D VAS, mm	67.6	11.44	59.5	9.41	0.018*	0.59
TUG, s	16.9	3.51	63.3	12.91	0.197	0.38
COP path, cm	21.9	10.13	22.7	11.46	0.741	0.10

*Table 1 shows the key to the abbreviations.*

p, probability associated with unpaired t-test or *Mann–Whitney U-test.

*d, Cohen's effect size.*

*Bold face highlights differences between the two groups at p < 0.05.*

*COP path, averaged across four standing conditions*.

## Results

Table 1 shows the descriptive data at baseline. Three patients in each group (*n* = 9) dropped out after 24 months. Figure 1 shows that 1, 5, and 9 patients died in the E + M, E, and C groups, respectively during the 72-month follow-up. Other than the 9 dropouts and 15 deaths, the attendance and compliance were 100% during the 3-week E-program and the 72-month follow-up. Excluding the baseline assessment, the 67 participants were followed-up 573 times in total (mean: 8.6/patient, range: 3–10). Before each E session and assessment, the therapists checked the exercise logs of the patients and did not detect the reports of adverse effects.

### Primary Outcome

**Figure 2:**
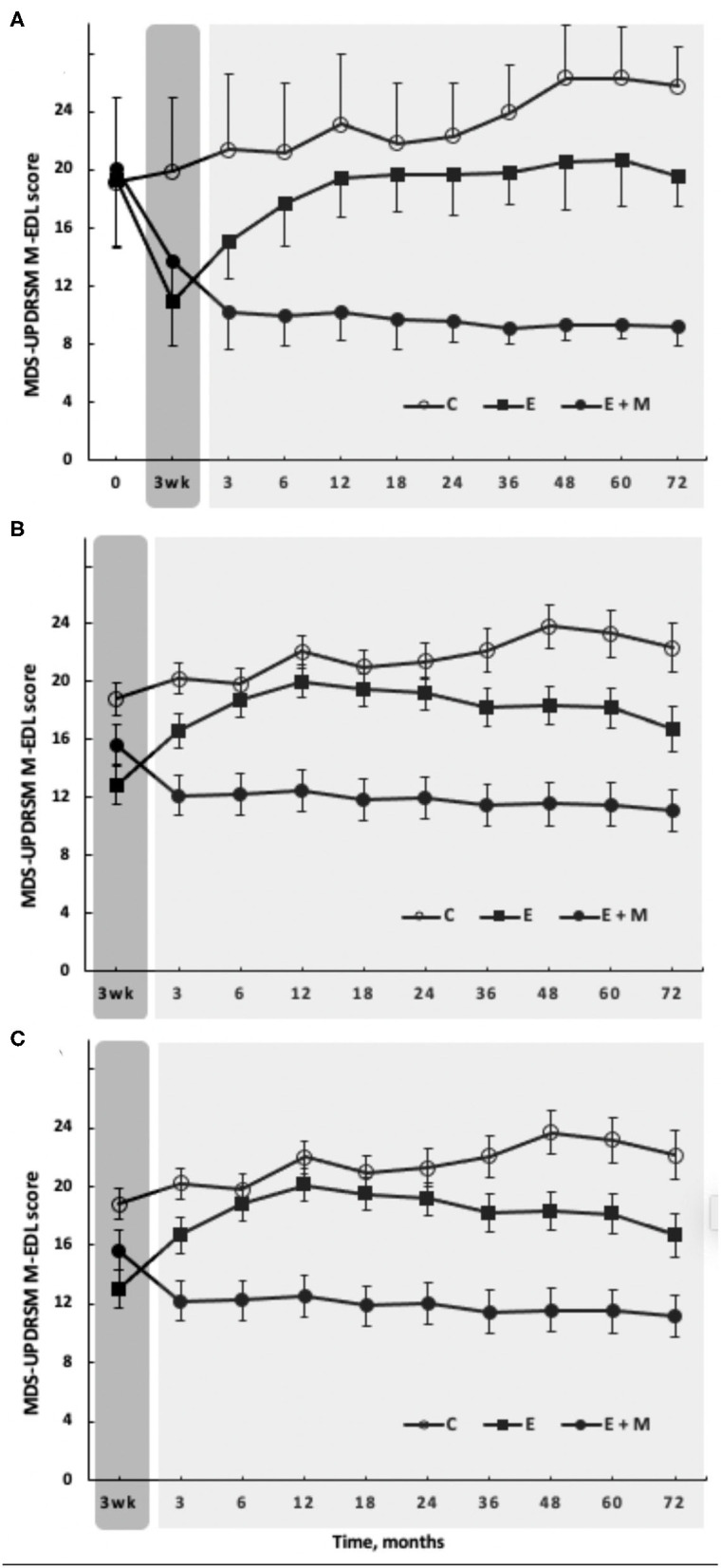
The effects of 3-week-long, high-intensity Exergaming agility exercise program (dark gray rectangle) with (filled circles, E + M, *n* = 19) or without (filled squares, E, *n* = 22) 72-month-long Exergaming agility exercise Maintenance program, on the primary outcome, M-EDL. The Control group (C, open circles, *n* = 26) performed no Exergaming agility exercise program and no Exergaming agility exercise Maintenance program. **(A)** Observed scores, such as baseline. **(B)** The longitudinal linear mixed effects modeling of M-EDL fitted with maximum likelihood estimation, adjusted for the baseline and 16 covariates. **(C)** The combined longitudinal linear mixed effects modeling with the semiparametric Cox regression to model survival. Time course trajectories based on the observed data **(A)** and the longitudinal linear mixed effects modeling **(B)** were robust so that adjusting the observed data for missing data (*n* = 3 per group starting at 24 months), deaths (*n* = 1, *n* = 5, and *n* = 9 in E + M, E, and C, respectively), the baseline differences, and covariates did not affect the treatment effects **(C)**. The vertical bars denote ±1 SD in **(A)** and 95% *CI* in **(B,C)**.

The likelihood ratio test, produced by the longitudinal linear mixed effects modeling, revealed a significant Group by Time interaction for M-EDL [chi^2^ (df:18) = 209.0, *p* < 0.001]. The 3-week E-program improved M-EDL by 6.3 points (±2.98; 30.2% ± 9.96) in E + M (*d* = 1.53) and by 8.4 points (±3.76; 42.7% ± 14.15) in E (*d* = 2.20, all *p* < 0.001). These changes exceeded the 0.7-point (±2.60; 6.0% ± 16.91) change in C (Figure 2A, left dark gray box).

After the E-program, the M-program in E+M further improved M-EDL for 3 months by 3.5 points (±2.09; 25.1% ± 13.49; *d* = 1.35, *p* < 0.001). After 3 months, the M-program did not further improve but sustained these initial gains until month 72 (Figure 2A, light gray box). In E, the effects of E-program lasted for 12 months, when the M-EDL levels returned to and remained at the baseline levels until month 72. The patients in the C group gradually worsened over 72 months by 8.5 points (±4.55; 56.3% ± 36.20; *d* = 1.43, *p* < 0.001). At 72 months, the difference in M-EDL between E + M vs. C was 16.6 points (*d* = 6.52), 10.4 points between E + M vs. E (*d* = 3.22), and 6.1 points between E vs. C (*d* = 2.21) in favor of E + M or E (Figure 2A, last data points on the right, all *p* < 0.001).

Compared with the results based on the observed data (Figure 2A), longitudinal linear mixed effects modeling adjusted for the baseline and covariates revealed a similar pattern of responses to the E-program and during follow-up in M-EDL (Figure 2B). The longitudinal linear mixed effects modeling combined with the survival analysis as a sensitivity analysis suggested that the time course trajectories based on the observed data and the mixed effects modeling were robust so that adjusting the observed data for missing data and deaths did not affect the treatment effects (Figure 2C).

### Secondary Outcomes

**Figure 3:**
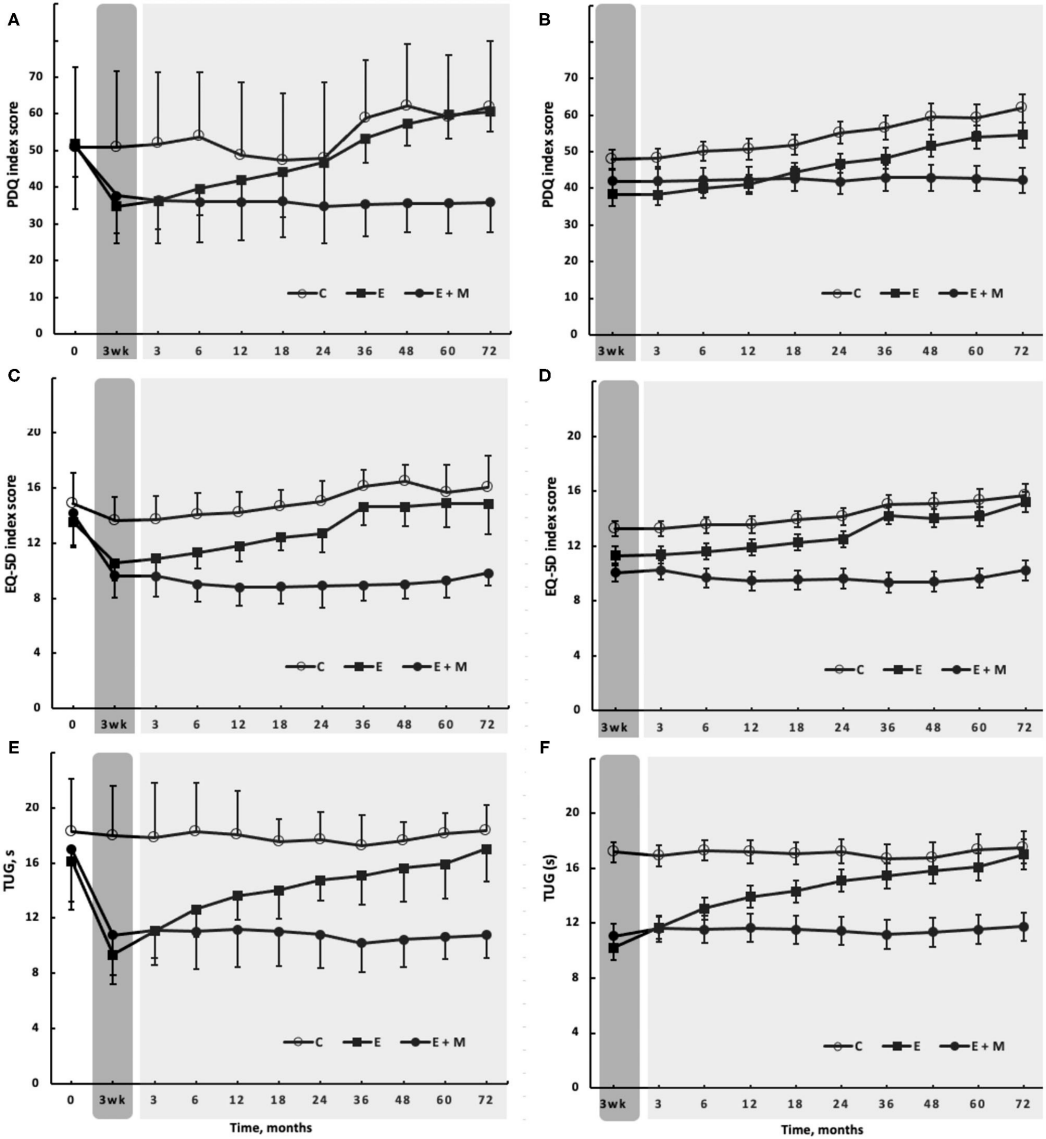
The effects of 3-week-long, high-intensity Exergaming agility exercise program (dark gray rectangle) with (filled circles, E + M, *n* = 19) or without (filled squares, E, *n* = 22) 72-month-long Exergaming agility exercise Maintenance program, on selected secondary outcomes, including: Parkinson's Disease Questionnaire- 39 [PDQ, **(A)**]; EuroQol five dimensions questionnaire on quality of life [EQ-5D, **(C)**], and Timed-Up-and-Go test [TUG, **(E)**]. **(A,C,D)** Show the observed scores, such as baseline. **(B,D,F)** Show the longitudinal linear mixed effects modeling of the corresponding outcomes fitted with maximum likelihood estimation, adjusted for the baseline and covariates.

The likelihood ratio from the longitudinal linear mixed effects modeling revealed a Group by Time interaction for BMI, PDQ, EQ-5D, EQ-5D VAS, TUG, BDI, and the four standing sway measures [chi^2^ range (df:18) = 32.2–117.3, *p* < 0.020 to *p* < 0.001] but not for SE [chi^2^ (df:18) = 26.5, *p* = 0.082]. There were no changes in any of the outcomes in C over the 3-week control period while E + M and E completed the E-program, but declined (*p* < 0.001) in all the variables by month 72 except in TUG (Figures 3E,F).

**Figure 4:**
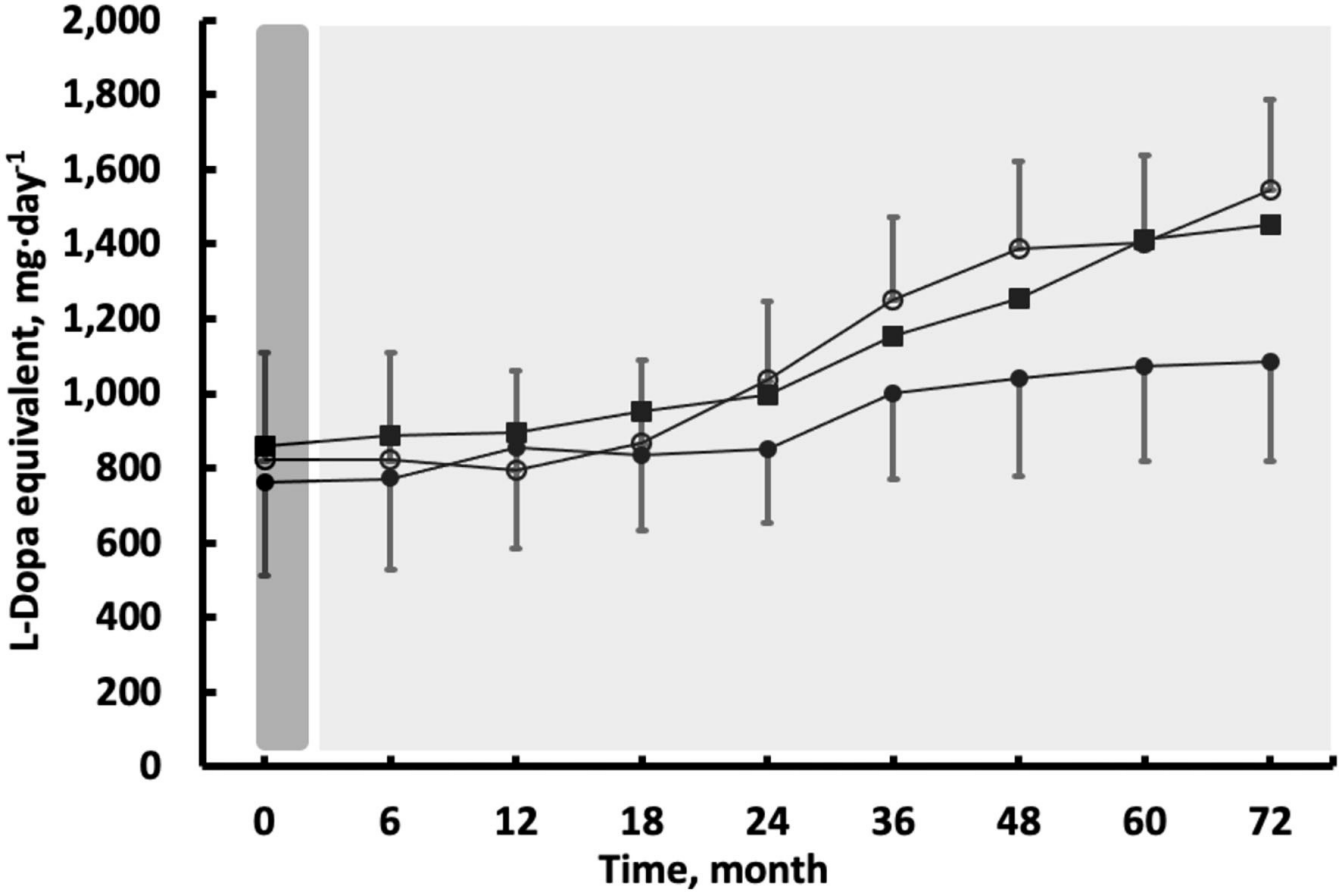
The effects of 3 weeks of high-intensity Exergaming with (E + M, filled circles) and without (E, filled squares) subsequent maintenance Exergaming program, or no exercise and no maintenance control (C, open circles) on L-dopa equivalent dose. Compared with C, the drug dose was lower in E + M between months 24 and 72. Compared with E, the drug dose was lower in E + M between months 48 and 72 (all *p* < 0.05). The vertical bars denote ±1 SD, omitted for clarity in the E group.

After the 3-week initial E-program, BMI remained unchanged (*p* > 0.05). By month 72, E + M lost 4.5 kg (±2.56; 5.6% ± 2.99; *d* = 0.82; *p* < 0.001), 2.9 and 3.7 kg more than C and E. Exergaming improved the PDQ similarly in E + M and E by 15.3 points (±10.84, *d* = 1.75; 30.3% ± 34.74). E+M maintained this improved score for 72 months, which was then ~25 points clinically better (*d* = 4.55) than the score in E or C (all *p* < 0.001, Figures 3A,B). The score of EQ-5D improved after Exergaming by 3.8 points (±14.71, 25.6% ± 20.27, *d* = 2.76, *p* < 0.001), with a difference of ~6 points (d = 3.21, *p* < 0.001) in favor of E + M vs. E and C at 72 months (Figures 3C,D). The EQ-5D and EQ-5D VAS (data not shown) revealed similar trajectories. Exergaming improved BDI by 2.7 points (±16.67, 14.6% ± 21.14, *d* = 4.48, *p* < 0.001) and E+M further improved BDI by 7.1 points (±4.79, 33.7% ± 16.76, *d* = 5.53, *p* < 0.001) at month 72. The effects of E-program lasted 36 months. At 72 months, the difference in BDI between E + M vs. C was 13.3 points (*d* = 5.92), 8.8 points between E + M vs. E (*d* = 2.247, and 4.6 points between E vs. C (*d* = 1.99) in favor of E + M or E (all *p* < 0.001). Exergaming improved TUG by 6.7 s (±18.26, 39.3% ± 26.38, *d* = 2.82, *p* < 0.001). The patients in the group E vs. group C completed TUG still ~5 s faster (*p* < 0.001, *d* = 1.33) at month 36 and E + M sustained its 6.7-s improvement and completed TUG ~7 s faster than the other two groups at month 72 (*p* < 0.001, *d* = 3.21, Figures 3E,F). Curiously, the TUG performance did not decline in C over 72 months. After Exergaming, sway path decreased by 11.1 cm (±41.87, 30.7% ± 36.83, *d* = 1.11, *p* < 0.001) in the four static standing tests. E + M maintained but did not improve this reduction at month 72 when sway path was ~20 cm shorter in E + M vs. E and C (*d* = 3.44, *p* < 0.001). Sway path in the four measures averaged, was still shorter by ~5 cm *(*d = 1.18, *p* < 0.001) in E vs. C at month 36. The L-Dopa equivalents increased least in E + M by 317.0 mg·d^−1^ (±301.05, 64.2% ± 68.50, *d* = 1.47,) compared with increases in E by 427.2 mg·d^−1^ (±208.67, 52.1% ± 26.24, *d* = 2.58) and in C by 765.2 mg·d^−1^ (336.76, 115.4% ± 69.45, *d* = 3.32; all the changes and differences *p* < 0.001, Figure 4).

As for the primary outcome, the observed (Figures 2, 3 left panels) and modeled data adjusted for the baseline and covariates (Figures 2, 3 right panels) revealed a similar pattern of responses to the E-program and during follow-up. The joint longitudinal and survival analysis as a sensitivity analysis also revealed that adjusting the observed data for missing data and deaths did not affect the treatment effects (data not shown).

### Correlation Analyses

The baseline M-EDL correlated with Exergaming-induced changes in M-EDL at 3 weeks *r* = −0.686 (*n* = 41) and at 72 months *r* = −0.696 (*n* = 29 completers, both *p* < 0.05). As the primary outcome in E + M plateaued after 3 months during the M-program (Figures 2A,B), we computed the correlations between the changes in M-EDL and changes up to 3 months in the secondary outcomes but found no associations (all *p* > 0.05).

### Characteristics of the Deceased Patients

Unrelated to the study, 1, 5, and 9 patients, died in E + M, E, and C groups, respectively (Figure 1). Cause of death was cerebrovascular disease (*n* = 5), neoplasms (*n* = 4), falls (*n* = 3), ischemic heart disease (*n* = 1), pulmonary embolism (*n* = 1), and PD (*n* = 1). Table 2 shows the baseline comparisons between the patients who died and those who were alive at month 72. At baseline, there were differences (*p* < 0.05) between these two groups in M-EDL and PD years.

## Discussion

We examined the immediate and lasting effects of a 3-week-long, high-intensity and high-frequency Exergaming agility E-program with and without a 6-year-long high-intensity Exergaming agility maintenance M-program, on the motor and clinical symptoms in PwPD. We found that the short-term E-program improved the PD symptoms up to a year during detraining but the subsequent 6-year-long M-program was needed to further increase or sustain the initial improvements in the symptoms, quality of life, and drug dose. The L-Dopa equivalents increased over 6 years but the increases were less in E+M than in the other two groups. The drug doses at the baseline and the changes in dosing did not correlate with the changes in other outcomes.

### Exergaming Effects on the Primary and Secondary Outcomes

The Exergaming E-program improved M-EDL by 7.4 points or 37% (Figure 2) above the 3.1-point clinically meaningful change (38). This is an important finding because M-EDL is an aggregate index of perceived and measured mobility, posture, and the clinical symptoms. The changes in M-EDL tend to exceed those reported previously after the aquatic and land-based programs lasting up to 12 months and of varying intensity (6, 14, 35, 47). Because the baseline M-EDL levels correlated strongly with the E-program induced gains in M-EDL, the patients with low baseline scores also tolerated the strong exercise stimulus well. We found no evidence in the M-EDL data that the program would be harmful for PwPD with HY stage 2–3. While not measured here, the current program elicits a heart rate of 80% age-predicted maximum, documenting high intensity (42). Whether such high intensity is actually necessary to improve M-EDL remains to be determined. The current changes indeed exceed the changes after low-intensity yoga, dance, and balance training reported in the reviews (2, 3, 17, 18). These changes in turn exceed the changes in M-EDL after very low-intensity physical and occupational therapy (48). Altogether, the data imply a dose-response relationship in M-EDL but such a relationship is not yet experimentally examined.

Except for BMI and SE, the E-program also substantially improved the secondary outcomes (*d* = 0.82–5.92, 14.6–39.3%) (Figure 3). PDQ measures the perception of well-being and the difficulties in performing activities of daily living of the patients. The 15.3-point improvement, well above the 4.7 clinically meaningful change (39), suggests that the E-program improved the life-outlook of the patients and reduced the impediments to execute daily tasks. The disease-specific improvements in PDQ were accompanied by the general health-related improvements indexed by EQ-5D (25.6%) and EQ-5D VAS. A depressive mood is a determinant of PD- and health-related QoL (11) and the E-program also improved the BDI substantially by 2.7 points (14.6%, *d* = 4.48). However, the changes in PDQ, EQ-5D, and BDI were not inter-related (all *p* > 0.05), providing no mechanistic links among the changes in these outcomes. Our BDI data strengthen the conclusion of a review reporting favorable effects of exercise on depression in PwPD (49). However, our data are not in line with another review, reporting mixed effects of exercise therapy on depression in PwPD and reporting also no heightened exercise-effects due to duration and intensity dose (11). The impairments in static and dynamic balance are the serious PD symptoms and the 39.3% and 30.7% reductions in TUG time (Figure 3) and standing sway path suggest the substantial favorable changes in these abilities. These changes exceed those reported previously after various forms of aquatic and land-based aerobic and weight-shift exercises (2, 3, 14, 17, 18, 47). To illustrate the potency of our 15-session, high-intensity multi-sensory exercise stimulus, the 6.7-s improvement in TUG (d = 2.82, *p* < 0.001, Figure 3) was 3-fold greater than the 2.2-s (*p* < 0.01) change reported after a 12-week-long, weight-shift-focused Qigong therapy (5).

### The Effects of Maintenance, Detraining, Symptom Progression, and Drug Dose on the Outcomes

To our knowledge, the present study, with 6 years, has the longest exercise maintenance program and detraining follow-ups in PwPD compared with the follow-up durations of 17 days, 1, 2, and 6 months (15, 50–52) or 2 years (23, 31, 32). While the complementary and alternative medical approaches are considered for minimizing the symptom-progression (12), exercise therapy is emerging as an efficacious symptom management option in PwPD (8, 53, 54). The uniform pattern produced by the M-program across the outcomes was that it further increased the exercise-induced gains for up to 3 months when these gains plateaued (Figures 2, 3). Thus, the M-program further improved the Exergaming-induced M-EDL gains by 3.5 points at 3 months (25.1%, *d* = 1.35, *p* < 0.001). Afterward, the three times weekly M-program sustained but did not further increase these gains at month 72 (Figure 2). A remarkable finding was that when the E-program was stopped, its effects on M-EDL lasted for 12 months (Figure 2), a pattern also observable in the secondary outcomes (Figure 3). Such lasting effects were also observed after 6 months of detraining following the rhythmical auditory stimuli-supported multimodal balance training (15).

Heterogeneity of the clinical symptoms makes the clinical evolution of PD non-linear. Without reliable markers of symptom-progression, the prediction of disease and symptom progression remains unknown (55, 56). In the present study, the rate of symptom progression in C varied between the outcomes but was linear, as higher order fits did not improve the relationship between the symptoms and time (data not shown). The reasons for linear symptom progression in the present study over 6 years to deviate from a non-linear progression over 9 years (57) could be that our follow-up was 3 years shorter and our patients had PD diagnosed 6.8 years prior to the start of the study. Thus, the initial fluctuations in the symptoms due to drug dosing and co-morbidities were absent (Table 1; Figures 2, 3). In this regard, an important finding was that 70% of the large differences in scores between E+M and C at 72 months (effect size range: 3.21–6.52) were caused not by the symptom worsening relative to the baseline in C but by the reductions in symptom deterioration in E + M. The clinicians should notice that TUG was not sensitive to mobility deterioration in our group of PwPD (Figure 3), an observation that agrees with the minimal (~1 s) decline in TUG over follow-ups of 3 months and 5 years (58, 59). Taken together, even a short-term but intensive E-program can moderate the motor and non-motor symptoms in PwPD but such improvements are transient. For lasting exercise-induced neuroprotective and restorative effects to occur, PwPD need to participate in a long-term exercise maintenance program (1–3, 8, 10, 11, 17, 18, 20, 60).

Concerning prediction of symptom progression and death, the follow-ups of PwPD for up to 38 years identified male sex, gait disorder, absence of rest tremor and asymmetry, age at diagnosis, disease subtype, cognitive status, and baseline motor score as the predictors (57, 61). Our data partially agree with some of these predictors of death, as PD years and M-EDL differed at the baseline between those who completed the study and those who have eventually deceased (Table 2). Because of the scarcity of such data, these data require confirmation.

Figure 4 shows that drug dose increased in the three groups but the increase was less in E+M starting at months 24 and 48 compared with C and E, respectively. These data partially agree with reductions in L-Dopa equivalent after two, 28-day multidisciplinary intensive rehabilitation treatments at 1-year interval (32). PwPD in the present study were at a more advanced stage of PD for a longer time and received a much higher drug those. The present long-term exercise data represented by E+M are in line with the speculation that long-term physical activity, especially at a high intensity, could slow symptom progression, reduce drug dose (Figure 4) (32), and ultimately reduce the risks for developing PD later in life (20, 62, 63).

## Limitations and Conclusions

The design lacked a group completing the M-program only without the initial E-program, making it unclear if the initial E-program potentiated the effects of M-program. The design also lacked a group that performed intensive, for example, cycling (42, 64) to determine the respective roles of fitness vs. sensorimotor stimuli in the improving quality of life and slow symptom-progression (7, 8, 10–12, 14, 58). Without a healthy age- and gender-matched control group, we were unable to parse the distinct effects of age and disease on the outcomes (65). C did not receive social attention for the times when E and E+M exercised in small groups. Because the patients exercised in small groups, the exercise stimulus was not set to the individual levels of disease severity (10). We conducted no separate testing “on” and “off” medication, to allow us to more precisely determine the effects of dopaminergic medication on mobility. As the assessments included only behavioral outcomes, we were unable to examine if the Exergaming agility E-and M-program induced neuroprotection and slowed neurodegeneration, mediating the behavioral changes (14). In addition, a narrow set of outcomes did not allow us to examine the underlying non-dopaminergic and cognitive mechanisms of mobility decline. We conducted the trial in a hospital supported by dedicated staff, a costly and complex system, prohibitive in many settings. Indeed, a home- vs. center-based E-program can improve the mobility similarly albeit not quality of life (66). We did not systematically examine the adverse events during the trial but the records suggest that the severe medical conditions unrelated to the trial caused the 15 deaths.

In conclusion, a short-term, high-intensity sensorimotor agility exercise program improved the PD symptoms up to a year during detraining but the subsequent 6-year-long maintenance program was needed to further increase or sustain the initial improvements in the symptoms, quality of life, and drug dose.

## Data Availability Statement

The original contributions presented in the study are included in the article/supplementary material, further inquiries can be directed to the corresponding author/s.

## Ethics Statement

The University Hospital's Ethics Committee approved the study protocol (IKEB2020/05) which was registered at https://clinicaltrials.gov/, Clinical Trial: NCT04559997. The patients/participants provided their written informed consent to participate in this study.

## Author Contributions

All authors listed have made a substantial, direct and intellectual contribution to the work, and approved it for publication.

## Conflict of Interest

The authors declare that the research was conducted in the absence of any commercial or financial relationships that could be construed as a potential conflict of interest.

## Publisher's Note

All claims expressed in this article are solely those of the authors and do not necessarily represent those of their affiliated organizations, or those of the publisher, the editors and the reviewers. Any product that may be evaluated in this article, or claim that may be made by its manufacturer, is not guaranteed or endorsed by the publisher.

## References

[1] Abbruzzese G Marchese R Avanzino L Pelosin E. Rehabilitation for Parkinson's disease: current outlook and future challenges. Parkinsonism Relat Disord. (2016) 22(Suppl. 1):S60–4. 10.1016/j.parkreldis.2015.09.005 26360239

[2] Bloem BR de Vries NM Ebersbach G. Nonpharmacological treatments for patients with Parkinson's disease. Mov Disord. (2015) 30:1504–20. 10.1002/mds.26363 26274930

[3] van der Kolk NM King LA. Effects of exercise on mobility in people with Parkinson's disease. Mov Disord. (2013) 28:1587–96. 10.1002/mds.25658 24132847

[4] Rodriguez-Oroz MC Moro E Krack P. Long-term outcomes of surgical therapies for Parkinson's disease. Mov Disord. (2012) 27:1718–28. 10.1002/mds.25214 23208668

[5] Wan Z Liu X Yang H Li F Yu L Li L. Effects of health qigong exercises on physical function on patients with Parkinson's disease. J Multidiscip Healthc. (2021) 14:941–50. 10.2147/JMDH.S303945 33953563 PMC8091455

[6] Vieira-Yano B Martini DN Horak FB de Lima-Pardini A Almeida F Santana VP. The adapted resistance training with instability randomized controlled trial for gait automaticity. Mov Disord. (2021) 36:152–63. 10.1002/mds.28298 32955752

[7] Rosenfeldt AB Koop MM Fernandez HH Alberts JL. High intensity aerobic exercise improves information processing and motor performance in individuals with Parkinson's disease. Exp Brain Res. (2021) 239:777–86. 10.1007/s00221-020-06009-0 33394100 PMC13040507

[8] Rafferty MR Nettnin E Goldman JG MacDonald J. Frameworks for Parkinson's disease rehabilitation addressing when, what, and how. Curr Neurol Neurosci Rep. (2021) 21:12. 10.1007/s11910-021-01096-0 33615420 PMC8215896

[9] Prusynski RA Gustavson AM Shrivastav SR Mroz TM. rehabilitation intensity and patient outcomes in skilled nursing facilities in the united states: a systematic review. Phys Ther. (2021) 101:pzaa230. 10.1093/ptj/pzaa230 33388761

[10] Martignon C Pedrinolla A Ruzzante F Giuriato G Laginestra FG Bouca-Machado R. Guidelines on exercise testing and prescription for patients at different stages of Parkinson's disease. Aging Clin Exp Res. (2021) 33:221–46. 10.1007/s40520-020-01612-1 32514871

[11] Dauwan M Begemann MJH Slot MIE Lee EHM Scheltens P Sommer IEC. Physical exercise improves quality of life, depressive symptoms, and cognition across chronic brain disorders: a transdiagnostic systematic review and meta-analysis of randomized controlled trials. J Neurol. (2021) 268:1222–46. 10.1007/s00415-019-09493-9 31414194 PMC7990819

[12] Church FC. Treatment options for motor and non-motor symptoms of parkinson's disease. Biomolecules. (2021) 11:612. 10.3390/biom11040612 33924103 PMC8074325

[13] Radder DLM Ligia Silva de Lima A Domingos J Keus SHJ van Nimwegen M Bloem BR. Physiotherapy in Parkinson's disease: a meta-analysis of present treatment modalities. Neurorehabil Neural Repair. (2020) 34:871–80. 10.1177/1545968320952799 32917125 PMC7564288

[14] Johansson H Hagstromer M Grooten WJA Franzen E. Exercise-Induced neuroplasticity in Parkinson's disease: a metasynthesis of the literature. Neural Plast. (2020) 2020:8961493. 10.1155/2020/8961493 32256559 PMC7079218

[15] Capato TTC de Vries NM IntHout J Barbosa ER Nonnekes J Bloem BR. Multimodal balance training supported by rhythmical auditory stimuli in Parkinson's disease: a randomized clinical trial. J Parkinsons Dis. (2020) 10:333–46. 10.3233/JPD-191752 31884492 PMC7029328

[16] Cancela JM Mollinedo I Montalvo S Vila Suarez ME. Effects of a high-intensity progressive-cycle program on quality of life and motor symptomatology in a Parkinson's disease population: a pilot randomized controlled trial. Rejuvenation Res. (2020) 23:508–15. 10.1089/rej.2019.2267 32336211

[17] Frazzitta G Balbi P Maestri R Bertotti G Boveri N Pezzoli G. The beneficial role of intensive exercise on Parkinson disease progression. Am J Phys Med Rehabil. (2013) 92:523–32. 10.1097/PHM.0b013e31828cd254 23552330

[18] Klamroth S Steib S Devan S Pfeifer K. Effects of exercise therapy on postural instability in parkinson disease: a meta-analysis. J Neurol Phys Ther. (2016) 40:3–14. 10.1097/NPT.0000000000000117 26655098

[19] Kwakkel G de Goede CJ van Wegen EE. Impact of physical therapy for Parkinson's disease: a critical review of the literature. Parkinsonism Relat Disord. (2007) 13(Suppl. 3):S478–87. 10.1016/S1353-8020(08)70053-1 18267287

[20] Olsson TT Svensson M Hallmarker U James S Deierborg T. Delayed clinical manifestation of Parkinson's disease among physically active: do participants in a long-distance ski race have a motor reserve? J Parkinsons Dis. (2021) 11:373. 10.3233/JPD-200004 32623406 PMC8090987

[21] Dijkstra BW Bekkers EMJ Gilat M de Rond V Hardwick RM Nieuwboer A. Functional neuroimaging of human postural control: a systematic review with meta-analysis. Neurosci Biobehav Rev. (2020) 115:351–62. 10.1016/j.neubiorev.2020.04.028 32407735

[22] Tollar J Nagy F Kovacs N Hortobagyi T. A high-intensity multicomponent agility intervention improves Parkinson patients' clinical and motor symptoms. Arch Phys Med Rehabil. (2018) 99:2478–84.e1. 10.1016/j.apmr.2018.05.007 29886075

[23] Tollar J Nagy F Kovacs N Hortobagyi T. Two-year agility maintenance training slows the progression of Parkinsonian symptoms. Med Sci Sports Exerc. (2019) 51:237–45. 10.1249/MSS.0000000000001793 30303934

[24] Steen Krawcyk R Vinther A Petersen NC Faber J Iversen HK Christensen T. Effect of home-based high-intensity interval training in patients with lacunar stroke: a randomized controlled trial. Front Neurol. (2019) 10:664. 10.3389/fneur.2019.00664 31316451 PMC6611174

[25] Orban A Garg B Sammi MK Bourdette DN Rooney WD Kuehl K. Effect of high-intensity exercise on multiple sclerosis function and phosphorous magnetic resonance spectroscopy outcomes. Med Sci Sports Exerc. (2019) 51:1380–6. 10.1249/MSS.0000000000001914 31205251 PMC6594188

[26] Schenkman M Moore CG Kohrt WM Hall DA Delitto A Comella CL. Effect of high-intensity treadmill exercise on motor symptoms in patients with *de novo* parkinson disease: a phase 2 randomized clinical trial. JAMA Neurol. (2017) 75:219–26. 10.1001/jamaneurol.2017.3517 29228079 PMC5838616

[27] Morberg BM Jensen J Bode M Wermuth L. The impact of high intensity physical training on motor and non-motor symptoms in patients with Parkinson's disease (PIP): a preliminary study. Neurorehabilitation. (2014) 35:291–8. 10.3233/NRE-141119 24990028

[28] Ahlskog JE. Does vigorous exercise have a neuroprotective effect in Parkinson disease? Neurology. (2011) 77:288–94. 10.1212/WNL.0b013e318225ab66 21768599 PMC3136051

[29] Conradsson D Lofgren N Nero H Hagstromer M Stahle A Lokk J. The effects of highly challenging balance training in elderly with Parkinson's disease: a randomized controlled trial. Neurorehabil Neural Repair. (2015) 29:827–36. 10.1177/1545968314567150 25608520 PMC4582836

[30] Pelosin E Avanzino L Barella R Bet C Magioncalda E Trompetto C. Treadmill training frequency influences walking improvement in subjects with Parkinson's disease: a randomized pilot study. Eur J Phys Rehabil Med. (2017) 53:201–8. 10.23736/S1973-9087.16.04301-X 27434611

[31] Corcos DM Robichaud JA David FJ Leurgans SE Vaillancourt DE Poon C. A two-year randomized controlled trial of progressive resistance exercise for Parkinson's disease. Mov Disord. (2013) 28:1230–40. 10.1002/mds.25380 23536417 PMC3701730

[32] Frazzitta G Maestri R Bertotti G Riboldazzi G Boveri N Perini M. Intensive rehabilitation treatment in early Parkinson's disease: a randomized pilot study with a 2-year follow-up. Neurorehabil Neural Repair. (2015) 29:123–31. 10.1177/1545968314542981 25038064

[33] Ascherio A Schwarzschild MA. The epidemiology of Parkinson's disease: risk factors and prevention. Lancet Neurol. (2016) 15:1257–72. 10.1016/S1474-4422(16)30230-7 27751556

[34] Heinzel S Lerche S Maetzler W Berg D. Global, yet incomplete overview of cohort studies in Parkinson's disease. J Parkinsons Dis. (2017) 7:423–32. 10.3233/JPD-171100 28582871

[35] Prodoehl J Rafferty MR David FJ Poon C Vaillancourt DE Comella CL. Two-year exercise program improves physical function in Parkinson's disease: the PRET-PD randomized clinical trial. Neurorehabil Neural Repair. (2015) 29:112–22. 10.1177/1545968314539732 24961994 PMC4276552

[36] Spiering BA Mujika I Sharp MA Foulis SA. Maintaining physical performance: the minimal dose of exercise needed to preserve endurance and strength over time. J Strength Cond Res. (2021) 35:1449–58. 10.1519/JSC.0000000000003964 33629972

[37] Horvath K Aschermann Z Acs P Bosnyak E Deli G Pal E. [Validation of the hungarian mds-updrs: why do we need a new parkinson scale?]. Ideggyogy Sz. (2014) 67:129–34. 26118257

[38] Horvath K Aschermann Z Kovacs M Makkos A Harmat M Janszky J. Minimal clinically important differences for the experiences of daily living parts of movement disorder society-sponsored unified Parkinson's disease rating scale. Mov Disord. (2017) 32:789–93. 10.1002/mds.26960 28218413

[39] Horvath K Aschermann Z Kovacs M Makkos A Harmat M Janszky J. Changes in quality of life in parkinson's disease: how large must they be to be relevant? Neuroepidemiology. (2017) 48:1–8. 10.1159/000455863 28161701

[40] Pompeu JE Arduini LA Botelho AR Fonseca MB Pompeu SM Torriani-Pasin C. Feasibility, safety and outcomes of playing Kinect Adventures! for people with Parkinson's disease: a pilot study. Physiotherapy. (2014) 100:162–8. 10.1016/j.physio.2013.10.003 24703891

[41] Tollar J Nagy F Csutoras B Prontvai N Nagy Z Torok K. High frequency and intensity rehabilitation in 641 subacute ischemic stroke patients. Arch Phys Med Rehabil. (2021) 102:9–18. 10.1016/j.apmr.2020.07.012 32861668

[42] Tollar J Nagy F Hortobagyi T. Vastly Different exercise programs similarly improve Parkinsonian symptoms: a randomized clinical trial. Gerontology. (2019) 65:120–7. 10.1159/000493127 30368495

[43] Stata. Stata Data Analysis and Statistical Software. 16th ed. College Station, TX: StataCorp LLC (2020).

[44] Raciborski R Yang X Marchenko Y. Joint Models of Longitudinal and Survival Data: Stata Package (2016).

[45] Diggle P Liang KY Zeger SL. Longitudinal Data Analysis. New York, NY: Oxford University Press (1994).

[46] Crowther MJ Abrams KR Lambert. PC. Joint modeling of longitudinal and survival data. Stata J. (2013) 13:165–84. 10.1177/1536867X1301300112

[47] Cugusi L Manca A Bergamin M Di Blasio A Monticone M Deriu F. Aquatic exercise improves motor impairments in people with Parkinson's disease, with similar or greater benefits than land-based exercise: a systematic review. J Physiother. (2019) 65:65–74. 10.1016/j.jphys.2019.02.003 30904467

[48] Clarke CE Patel S Ives N Rick CE Woolley R Wheatley K. Clinical effectiveness and cost-effectiveness of physiotherapy and occupational therapy versus no therapy in mild to moderate Parkinson's disease: a large pragmatic randomised controlled trial (PD REHAB). Health Technol Assess. (2016) 20:1–96. 10.3310/hta20630 27580669 PMC5018686

[49] Wu PL Lee M Huang TT. Effectiveness of physical activity on patients with depression and Parkinson's disease: A systematic review. PLoS One. (2017) 12:e0181515. 10.1371/journal.pone.0181515 28749970 PMC5531507

[50] Perez de la Cruz S. Effectiveness of aquatic therapy for the control of pain and increased functionality in people with Parkinson's disease: a randomized clinical trial. Eur J Phys Rehabil Med. (2017) 53:825–32. 10.23736/S1973-9087.17.04647-0 28627861

[51] Vivas J Arias P Cudeiro J. Aquatic therapy versus conventional land-based therapy for Parkinson's disease: an open-label pilot study. Arch Phys Med Rehabil. (2011) 92:1202–10. 10.1016/j.apmr.2011.03.017 21807139

[52] Volpe D Giantin MG Manuela P Filippetto C Pelosin E Abbruzzese G. Water-based vs. non-water-based physiotherapy for rehabilitation of postural deformities in Parkinson's disease: a randomized controlled pilot study. Clin Rehabil. (2017) 31:1107–15. 10.1177/0269215516664122 27512099

[53] Oliveira de Carvalho A Filho ASS Murillo-Rodriguez E Rocha NB Carta MG Machado S. Physical exercise for Parkinson's disease: clinical and experimental evidence. Clin Pract Epidemiol Ment Health. (2018) 14:89–98. 10.2174/1745017901814010089 29785199 PMC5897963

[54] Xu X Fu Z Le W. Exercise and Parkinson's disease. Int Rev Neurobiol. (2019) 147:45–74. 10.1016/bs.irn.2019.06.003 31607362

[55] Ma LY Tian Y Pan CR Chen ZL Ling Y Ren K. Motor progression in early-stage Parkinson's disease: a clinical prediction model and the role of cerebrospinal fluid biomarkers. Front Aging Neurosci. (2020) 12:627199. 10.3389/fnagi.2020.627199 33568988 PMC7868416

[56] Cilia R Cereda E Akpalu A Sarfo FS Cham M Laryea R. Natural history of motor symptoms in Parkinson's disease and the long-duration response to levodopa. Brain. (2020) 143:2490–501. 10.1093/brain/awaa181 32844196 PMC7566883

[57] Reinoso G Allen JC Jr Au WL Seah SH Tay KY Tan LC. Clinical evolution of Parkinson's disease and prognostic factors affecting motor progression: 9-year follow-up study. Eur J Neurol. (2015) 22:457–63. 10.1111/ene.12476 24888502

[58] Johansson C Lindstrom B Forsgren L Johansson GM. Balance and mobility in patients with newly diagnosed Parkinson's disease – a five-year follow-up of a cohort in northern Sweden. Disabil Rehabil. (2020) 42:770–8. 10.1080/09638288.2018.1509240 30451551

[59] Silva AZD Israel VL. Effects of dual-task aquatic exercises on functional mobility, balance and gait of individuals with Parkinson's disease: a randomized clinical trial with a 3-month follow-up. Complement Ther Med. (2019) 42:119–24. 10.1016/j.ctim.2018.10.023 30670228

[60] Tomlinson CL Herd CP Clarke CE Meek C Patel S Stowe R. Physiotherapy for Parkinson's disease: a comparison of techniques. Cochrane Database Syst Rev. (2014) 2014:CD002815. 10.1002/14651858.CD002815.pub2 24936965 PMC7120367

[61] Pinter B Diem-Zangerl A Wenning GK Scherfler C Oberaigner W Seppi K. Mortality in Parkinson's disease: a 38-year follow-up study. Mov Disord. (2015) 30:266–9. 10.1002/mds.26060 25447933

[62] Thacker EL Chen H Patel AV McCullough ML Calle EE Thun MJ. Recreational physical activity and risk of Parkinson's disease. Mov Disord. (2008) 23:69–74. 10.1002/mds.21772 17960818 PMC2387117

[63] Xu Q Park Y Huang X Hollenbeck A Blair A Schatzkin A. Physical activities and future risk of Parkinson disease. Neurology. (2010) 75:341–8. 10.1212/WNL.0b013e3181ea1597 20660864 PMC2918886

[64] Tollár J Nagy F BE TO K TO Szita K CsutorAs B. exercise effects on multiple sclerosis quality of life and clinical-motor symptoms. Med Sci Sports Exerc. (2020) 52:1007–14. 10.1249/MSS.0000000000002228 31876670

[65] Wilson J Alcock L Yarnall AJ Lord S Lawson RA Morris R. Gait progression over 6 years in parkinson's disease: effects of age, medication, and pathology. Front Aging Neurosci. (2020) 12:577435. 10.3389/fnagi.2020.577435 33192470 PMC7593770

[66] Flynn A Allen NE Dennis S Canning CG Preston E. Home-based prescribed exercise improves balance-related activities in people with Parkinson's disease and has benefits similar to centre-based exercise: a systematic review. J Physiother. (2019) 65:189–99. 10.1016/j.jphys.2019.08.003 31521554

